# What do You Need to Know after Diabetes and before Diabetic Retinopathy?

**DOI:** 10.14336/AD.2025.0289

**Published:** 2026-04-30

**Authors:** Shiyu Zhang, Jia Liu, Heng Zhao, Yuan Gao, Changhong Ren, Xuxiang Zhang

**Affiliations:** ^1^Department of Ophthalmology, Xuanwu Hospital, Capital Medical University, Beijing, China.; ^2^Beijing Institute of Brain Disorders, Laboratory of Brain Disorders, Laboratory for Clinical Medicine, Ministry of Science and Technology, Collaborative Innovation Center for Brain Disorders, Beijing Advanced Innovation Center for Big Data-based Precision Medicine, Capital Medical University, Beijing, China.; ^3^Beijing Key Laboratory of Hypoxia Translational Medicine, Xuanwu Hospital, Center of Stroke, Beijing Institute of Brain Disorder, Capital Medical University, Beijing, China.

**Keywords:** Diabetic Retinopathy, Retinal Endothelial Cell, Biomarkers, Intervention, Early Detection

## Abstract

Diabetic retinopathy (DR) is a leading cause of vision impairment and blindness among individuals with diabetes mellitus. Current clinical diagnostic criteria mainly base on visible vascular structure changes, which are insufficient to identify diabetic patients without clinical DR (NDR) but with dysfunctional retinopathy. This review focuses on retinal endothelial cells (RECs), the first cells to sense and respond to elevated blood glucose. As blood glucose rises, RECs undergo compensatory and transitional phases, and the correspondingly altered molecules are likely to become biomarkers and targets for early prediction and treatment of NDR with dysfunctional retinopathy. This article elaborated the possible pathophysiological processes focusing on RECs and summarized recently published and reliable biomarkers for early screening and emerging intervention strategies for NDR patients with dysfunctional retinopathy. Additionally, references for clinical medication selection and lifestyle recommendations for this population are provided. This review aims to deepen the understanding of REC biology and NDR pathophysiology, emphasizes the importance of early detection and intervention, and points out future directions to improve the diagnosis and treatment of NDR with dysfunctional retinopathy and to reduce the occurrence of DR.

## 1. Introduction

Diabetic Retinopathy (DR) is one of the most common microvascular complications of diabetes mellitus and a leading cause of visual impairment and vision loss worldwide. The International Diabetes Federation projects that by 2045, 783.2 million people globally will be diagnosed with diabetes mellitus [1], highlighting the escalating global health challenge posed by DR and the critical need for its early identification and intervention. A large cross-sectional study conducted in Denmark in 2017 found that 35% of individuals newly diagnosed with type 2 diabetes already presented with complications [2], and approximately 10% had already developed DR [3, 4]. This finding suggests that a significant proportion of patients miss the optimal window for early intervention by the time of their diabetes diagnosis.

Currently, DR severity is typically classified using the modified Airlie House classification scale, as applied in the Early Treatment Diabetic Retinopathy Study (ETDRS), which is based on the presence and severity of microvascular abnormalities [5]. Clinically, DR is categorized into nonproliferative DR (NPDR) and proliferative DR (PDR), characterized primarily by morphological vascular abnormalities and pathological neovascularization, respectively. While the precise causal relationship between retinal vasculopathy and neuropathy in DR remains debated, studies suggesting that neuropathy precedes detectable vascular lesions often rely on current diagnostic criteria [6]. These criteria primarily assess vascular morphology and may not adequately capture early indicators of vascular dysfunction.

Consequently, current clinical diagnostic criteria are often inadequate for identifying patients who lack clinically apparent DR (termed NDR) but exhibit underlying dysfunctional retinopathy. Traditionally, NDR denotes the absence of clinically visible retinopathy; however, this definition fails to encompass patients experiencing solely functional deficits. This stage of dysfunctional retinopathy likely represents the most opportune time for early intervention. These limitations in diagnostic criteria contribute to delayed diagnosis, often occurring only after irreversible visual impairment has developed, thereby missing the critical window for preventative treatment. Indeed, some individuals classified as NDR exhibit subtle pathological changes, such as decreased vascular density (VD) in the deep retinal layers, enlargement of the foveal avascular zone (FAZ), and structural or electrophysiological abnormalities of the retina (reviewed below). This review specifically focuses on this NDR population exhibiting functional retinopathy.

**Figure 1.**
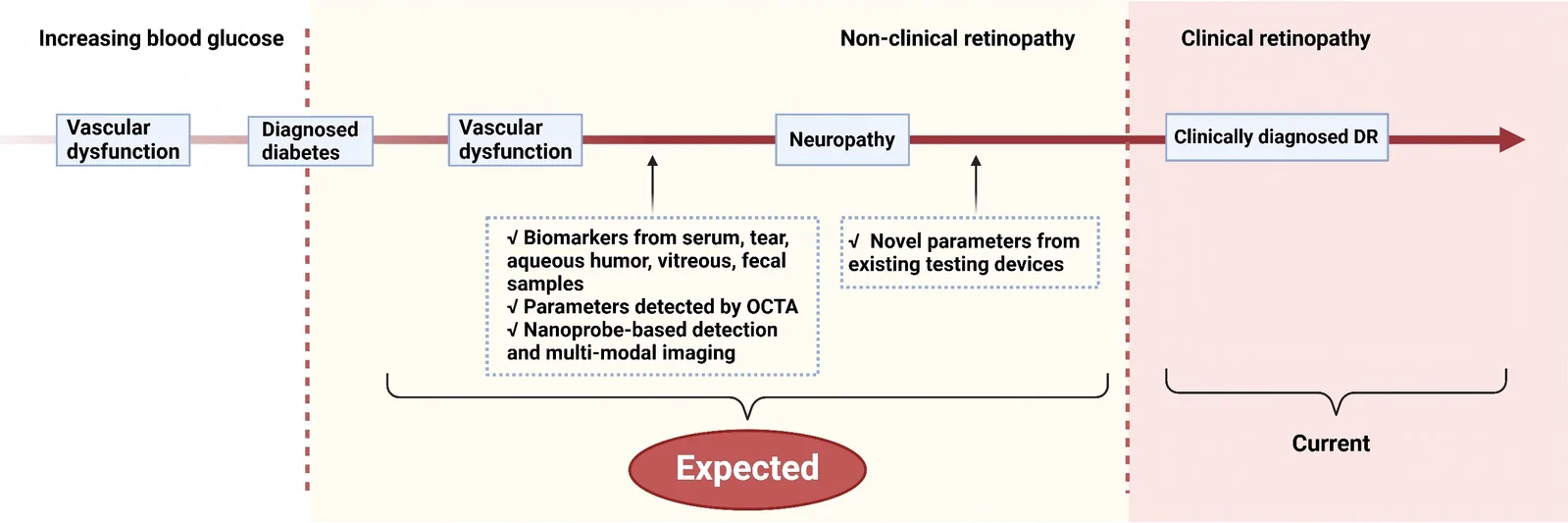
**The timeline of DR**. Vascular dysfunction and neuropathy are prior to clinical DR, which are expected diagnostic and preventive period, but lack of evidence to identify these lesions. DR: diabetic retinopathy.

The precise retinal pathological changes occurring during this dysfunctional retinopathy stage in NDR patients remain poorly understood. Consequently, reliable biomarkers for screening and targeted preventive measures are lacking. While this population is typically advised to maintain healthy lifestyle behaviors and often receives multiple systemic medications (e.g., hypoglycemic, lipid-lowering, antihypertensive, and antiplatelet agents), the specific impact of these interventions on the progression from dysfunctional retinopathy to clinically apparent DR is not fully elucidated. These knowledge gaps underscore the urgent need for better understanding of dysfunctional retinopathy period in NDR patients. Such understanding is crucial for developing more sensitive and specific predictive methods and intervention strategies, including, but not limited to, the identification of biomarkers for early detection and targeted therapy (Fig. 1).

**Figure 2.**
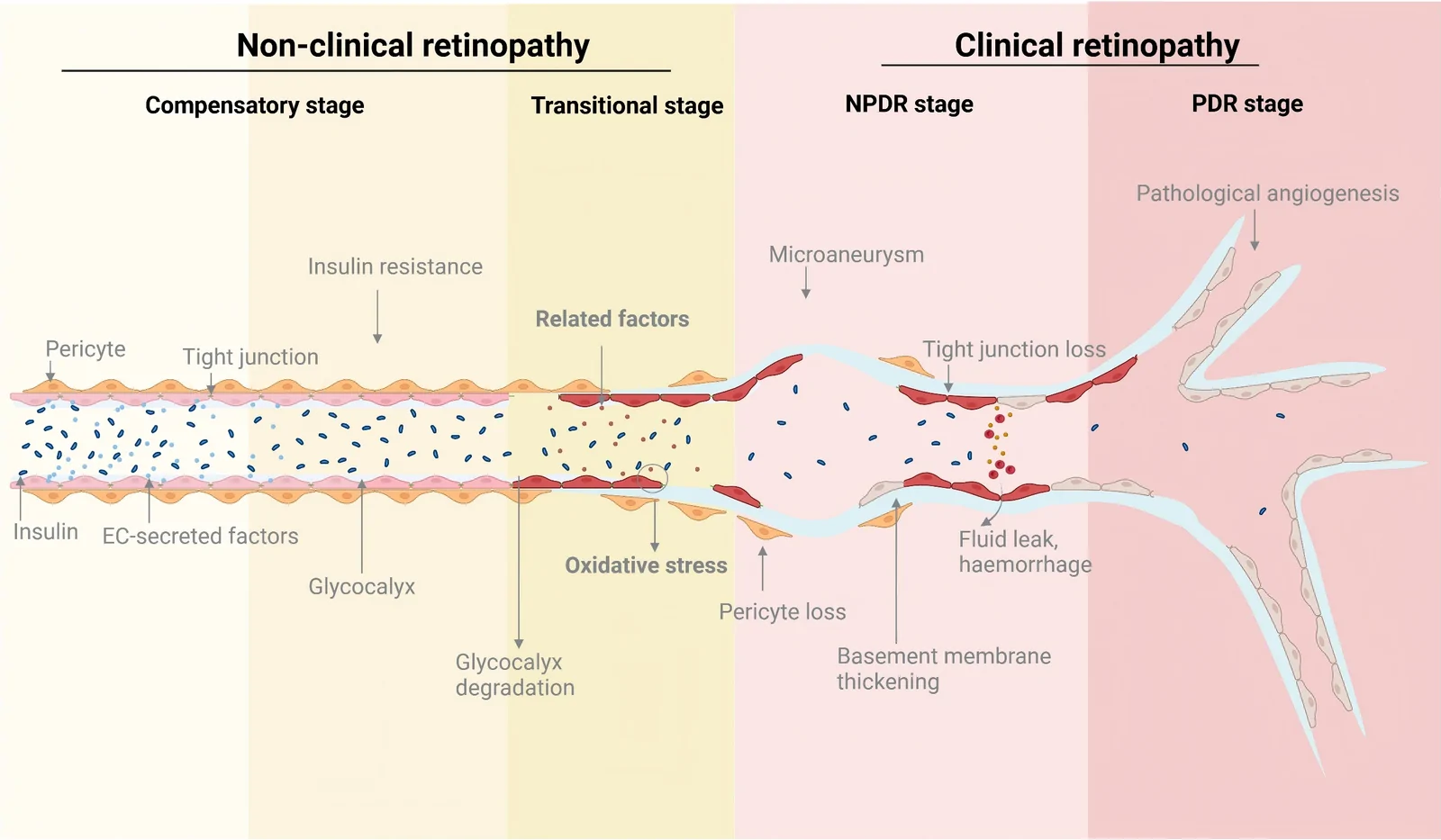
**Schematic diagram of REC physiological and pathological alterations**. Before clinically visible vasculopathy, vessels undergo functional changes. REC is the first cell type exposure to hyperglycemia so that better understanding of REC pathophysiological process helps early identification and intervention. REC: retinal endothelial cell.

This article reviews the alterations in retinal endothelial cells (RECs) that correspond with vascular pathological development during the NDR stage, particularly focusing on the period of dysfunctional retinopathy. RECs, as the principal component of retinal vasculature, are among the first cell types to sense and respond to elevated glucose levels. Their subsequent adaptive changes and eventual dysfunction play a pivotal role in the pathogenesis of DR (Fig. 2) [7]. Unlike the current reviews on preclinical DR or REC dysfunction, this review synthesizes current knowledge on the pathophysiological progression during the NDR stage specifically from the perspective of REC alterations. This approach aims to provide novel insights relevant to screening strategies, patient self-management, and early medical intervention for NDR patients experiencing dysfunctional retinopathy.

## 2. Pathophysiological changes before visible vascular abnormalities

### 2.1 The compensatory stage

**Figure 3.**
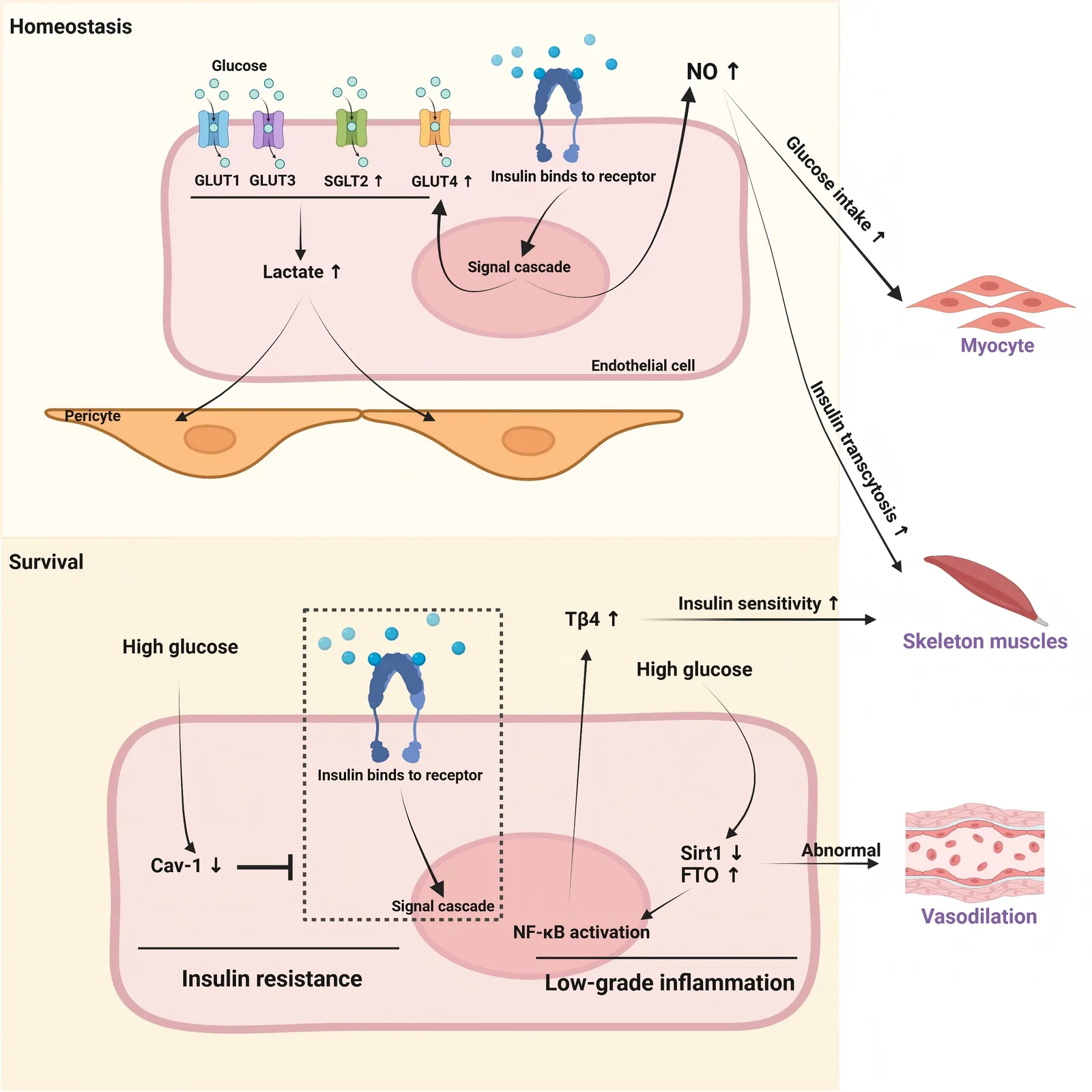
**Schematic diagram of REC compensatory period**. Faced with increasing blood glucose, REC has regulated homeostasis by directly and indirectly enhancing glucose intake. Then REC undergoes survival mechanisms to reduce high glucose-induced metabolic disorders. Cav-1: caveolin-1; FTO: fat mass and obesity-associated protein; GLUT: glucose transporter; SGLT2: sodium-glucose cotransporter 2; Sirt1: sirtuin1; Tβ4: thymosin beta-4.

During the initial phase following the onset of hyperglycemia, RECs initiate compensatory mechanisms. These adaptations aim to manage the elevated glucose overload, maintain vascular homeostasis and retinal health, and ultimately ensure cell survival amidst glucose toxicity (Fig. 3).

#### 2.1.1 Regulated homeostasis

RECs express several passive glucose transporters (GLUTs), including GLUT1, GLUT3, and the insulin-dependent GLUT4. In hyperglycemic conditions, the activity of these transporters leads to increased glucose influx into endothelial cells (ECs). Upon entry, a portion of this glucose is phosphorylated to glucose-6-phosphate [8] to enter the glycolytic pathway. Glycolysis serves as the predominant bioenergetic pathway for ECs, generating up to 85% of their total cellular ATP content [9]. Nearly 90% of the glucose entering ECs subsequently exits the cells as lactate [10]. The endothelium-derived lactate is recognized as an important energy substrate for neighboring pericytes; its deficiency can lead to pericyte loss from the vascular wall and consequently impair blood-retinal barrier (BRB) function [11]. However, excessive lactate accumulation is detrimental, as it can induce proteolytic cleavage of VE-cadherin, leading to the enhanced endocytosis in ECs and subsequent BRB breakdown [12].

Beyond GLUTs, sodium-glucose cotransporters (SGLTs) also play a role in glucose uptake. Under hyperglycemia conditions, ECs upregulate SGLT2 mRNA expression and protein synthesis, thereby increasing glucose uptake. The effect can’t be observed under normoglycemic conditions [8, 13].

Insulin signaling also plays a role in this stage. Insulin stimulation of ECs significantly elevates the expression and activity of endothelial nitric oxide synthase (eNOS), the enzyme responsible for generating nitric oxide (NO) [14]. NO, in turn, may enhance insulin transcytosis from circulation into the interstitium of skeletal muscle [15] and facilitate insulin-stimulated capillary recruitment, vasodilation and increased blood flow, thereby promoting glucose uptake by myocytes [14].

#### 2.1.2 Survival mechanisms

To cope with increased metabolic demands and high glucose-induced stress, RECs initiate adaptive survival mechanisms. Research shows that ECs may develop insulin resistance earlier than other tissues [15], indicating that insulin resistance could represent an early survival strategy. Insulin resistance is characterized as a state of reduced responsiveness to insulin in target cells or tissues [16, 17].

Caveolin-1 (Cav-1), the primary membrane protein of caveolae in ECs, plays a crucial role in regulating insulin receptor levels and signaling. It is particularly enriched in the peripheral and cerebral vasculature [18–20]. Studies have reported decreased Cav-1 levels in the cerebral microvessels of diabetic mice [18], suggesting that depletion of Cav-1 may contribute to endothelial insulin resistance. While direct evidence for Cav-1 alterations in RECs under high glucose is currently limited, pathological changes of the blood-brain barrier in diabetes are often parallel those in the BRB [21, 22]. Therefore, it is plausible that RECs also develop insulin resistance potentially linked to reduced Cav-1 expression in diabetes. Paradoxically, while reduced Cav-1 might be expected to decrease transcytosis and thus enhance barrier integrity [23], studies indicate that Cav-1 reduction can actually induce BRB permeability, suggesting that only adequate Cav-1 levels can maintain the integrity of the neurovascular unit [24, 25]. Furthermore, reduced Cav-1 levels have been associated with decreased EC senescence [26], implying a complex role where Cav-1 downregulation may offer cellular advantages despite its potential negative impact on barrier function and insulin signaling.

Besides mechanisms involving Cav-1, ECs can limit insulin responsiveness by modulating NO production. Endothelial Sirtuin1 (Sirt1) levels are reportedly diminished in diabetic animal models [27]. Within the vascular endothelium, Sirt1 regulates NO production and lower NO levels in turn restrict insulin transcytosis and hypoglycemic effect. Additionally, the RNA demethylase FTO (fat mass and obesity-associated protein) appears involved. While loss of endothelial FTO preserves the vasodilatory response to insulin under high-fat diet condition, FTO expression significantly increases in a dose-dependent manner in human retinal microvascular endothelial cells (HRMECs) treated with high glucose [28].

**Figure 4.**
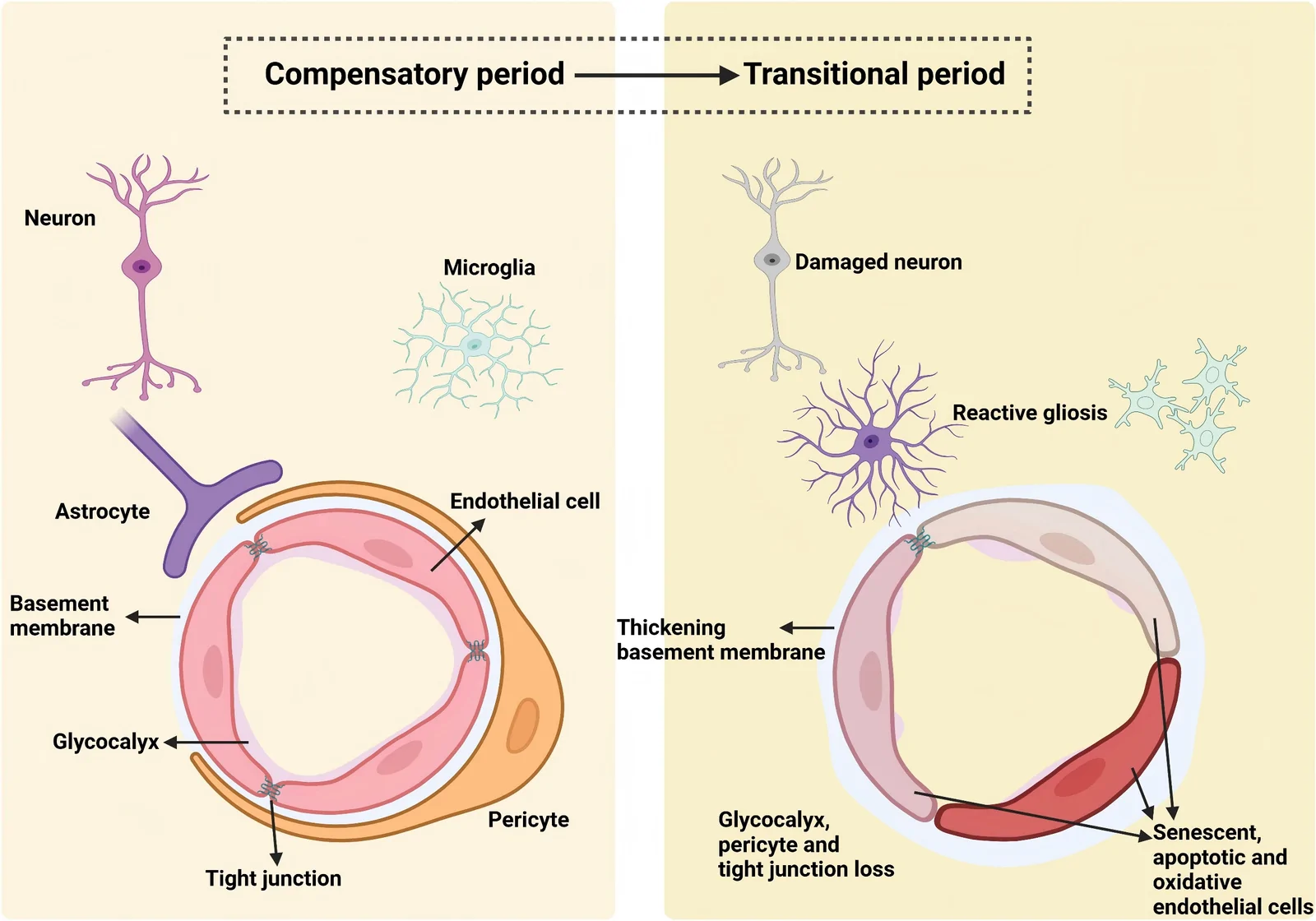
**Schematic diagram of REC transitional period**. Prolonged high glucose contributes to a series of metabolic disorders, which forms the pathophysiological basis of NPDR.

Low-grade inflammation may represent another complex adaptive or survival response. Under high glucose, endothelial Sirt1 deficiency contributes to the activation of the transcription factor NF-kB, promoting a pro-inflammatory state [29]. Similarly, upregulated FTO also contributes to activated NF-kB and inflammation in a glucose dose-dependent manner, potentially via RNA demethylation of *Tnip1*, referred to as the direct effector of FTO and the negative regulator of NF-kB [28]. Activated NF-kB can, in turn, upregulate the expression and secretion of thymosin beta-4 (Tβ4). Tβ4 is a G-actin-sequestering protein with pleiotropic effects, exerting tissue repair as well as anti-inflammatory effects. Tβ4 is abundantly expressed in ECs, and its release into circulation has been shown to enhance skeletal muscle insulin sensitivity [29], suggesting a possible systemic compensatory role originating from the endothelium.

### 2.2 The transitional stage

As hyperglycemia persists, REC function enters a stage of decompensation. The stage is characterized by a cascade of pathological changes, including thickening of the basement membrane (BM), disrupted crosstalk with pericytes and glial cells, loss of tight junction (TJ) protein, degradation of the glycocalyx, enhancement of endothelial-to-mesenchymal transition (EndoMT) and finally REC senescence and apoptosis. This series of pathological changes forms the structural basis for vascular lesions observed during the NPDR period (Fig. 4).

#### 2.2.1 Basement membrane thickening

Collagen type IV (Col IV), primarily produced by ECs, is the main component of the vascular BM [30, 31]. Both *in vivo* and *in vitro* evidence shows increased synthesis of Col IV by RECs under high glucose conditions [30, 32, 33]. Although studies have reported that urinary Col IV levels do not differ significantly between patients with DR and healthy individuals [33], factors contributing to the increased expressions of Col IV may serve as predictors of early disease progression. For instance, increased glucocorticoids can promote Col IV remodeling and elevated glucocorticoid levels have been correlated with the presence and severity of DR [32], indicating their potential as early biomarkers. However, given their inherent circadian rhythm, further research is required to validate this potential. Osteopontin (OPN), a multifunctional extracellular matrix glycoprotein, is present at higher levels in the vitreous fluid of patients with DR compared to those without DR. OPN can upregulate Col IV expression by suppressing miR-29a in RECs [30]. While serum and urinary OPN levels have been reported independent correlation with diabetic cardiomyopathy and diabetic kidney diseases in early stage of diabetes [34, 35], studies investigating relationship between OPN and early DR remain limited.

#### 2.2.2 Disordered interaction of EC and pericytes

Platelet-derived growth factor B (PDGF-B)/PDGF receptor β (PDGFRβ) signaling is recognized as indispensable for EC-pericyte interaction [36, 37]. However, Park et al. reported that while PDGF-B/PDGFRβ signaling is indispensable in retinal growing vessels, Tie2/Akt signaling is more crucial for maintaining interactions in stable vessels. This indicates that dysregulation of Tie2/Akt signaling may play predominant roles in early DR, which typically lacks neovascularization [37]. These investigators pointed out that Tie2 shedding from RECs might occur immediately after pericyte dropout [37], implying that shed Tie2 could serve as an indicator for pericyte dropout and potentially as a sensitive biomarker. However, the initial levels of shedding Tie2 may be too low to detect with current methods, becoming measurable only after visible vascular alterations.

Apart from direct cell-cell interactions, communication via extracellular vesicles, including those containing circular RNAs (circRNAs), has been reported the important roles in EC-pericyte crosstalk. *In vivo* and *in vitro* experiments demonstrate that high glucose can promote circular RNA-cPWWP2A expression in pericytes (but not RECs), and this circRNA can be transferred to RECs. Within RECs, cPWW2A acts as the molecular sponge for miR-579, thereby indirectly upregulating occludin1 and Sirt1 expression and modulating EC-pericyte crosstalk [38]. However, the transport of extracellular vesicles from pericytes to RECs may be hindered by the thickening BM, potentially impairing this mode of intercellular interactions of EC-pericyte.

In addition, under high glucose stress, the transcription of another circRNA, ZNF532, is also upregulated in pericytes. Although cZNF532 can promote cyclin-dependent kinase 2(CDK2) under normal conditions, overexpressed cZNF532 can exert repressive effect on CDK2 [39]. Overexpressed cZNF532 can be seen as a sink of miR-29a-3p, thereby modulating CDK2 activity. CDK2 is a serine/threonine protein kinase involved in cell cycle regulation that has been implicated in DR, with its expression tightly linked to cell proliferation [39–41]. In RECs, elevated levels of FTO (induced by increased lactate resulting from hyperglycemia) can promote increased CDK2 expression. Elevated FTO in RECs can subsequently attenuate pericyte coverage rate and disrupt EC-pericyte crosstalk. The disruption may be linked to EC connection failures associated with abnormal proliferation (potentially involving upregulated CDK2 activity) [40]. Therefore, unbalanced CDK2 production and cell proliferation in RECs and pericytes are likely to contribute to pericyte loss and abnormal EC-pericyte crosstalk.

#### 2.2.3 Glycocalyx degradation

Retina is an immune-privileged site, and the alterations in BRB permeability allow the infiltration of peripheral immune cells [16]. Endothelial glycocalyx, a layer coating the luminal surface of ECs, forms part of the endothelial barrier and acts as the first line of defense, limiting entry and infiltration of peripheral immune cells [42, 43]. Studies have reported systemic glycocalyx damage, a reduction in glycocalyx thickness preceding observable diabetic vascular abnormalities, and decreased levels of glycocalyx in human retinal microvascular endothelial cells (HRMEC) treated with high glucose [44–46]. Thus, a better understanding of endothelial glycocalyx facilitates identifying DR earlier.

Endothelial glycocalyx is a dynamic structure of proteoglycans and glycosaminoglycans (GAGs) [43]. Proteoglycans are decorated with GAG chains, primarily heparan sulfate (HS), chondroitin sulfate (CS), and hyaluronic acid (HA) [47]. While CS is the dominant GAG in rat RMEC and retina, followed by HS [46], HS appears to be the key component of the mouse retinal microvascular barrier [48]. Given the research by Cutler et al. did not observe significant differences in CS between DR patients and normal subjects [49], HS may be the most functionally relevant GAG component in the pathophysiology of DR, although the impairment mechanisms haven’t been clear yet.

Molecules secreted from RECs can directly contribute to glycocalyx degradation. Li et al. reported that skin ECs in psoriasis secret insulin-like growth factor binding protein-7 (IGFBP7), which directly binds to HS, thereby degrading the spatial structure of the endothelial glycocalyx, exposing underlying adhesion molecules, and driving T cells extravasation [50]. IGFBP7 is considered as the EC biomarker and a component of the senescence-associated secretory phenotype (SASP) (discussed further below) [51]. Since senescent RECs are present in early stage of DR [52], IGFBP7 holds potential as an early biomarker. However, its sensitivity still requires further exploration, particularly concerning the temporal relationship between REC senescence and glycocalyx degradation. EC-derived enzymes also play an important role in HS degradation. Heparanase, an endogenous endo-β-D-glucuronidase with high specificity for HS, is predominantly produced by ECs. Recently, it was reported that sepsis-induced lactate increases the lactylation of K18 of histone H3 (H3K18la), which is enriched at the promoter of the key heparanase transcription factor, leading to upregulated secretion of heparanase in pulmonary microvascular ECs [53]. Increased lactate and H3K18la have been validated in DR [40], suggesting a potential mechanism for increased heparanase activity in the diabetic retina.

Some molecules may indirectly aggravate glycocalyx damage by inducing immune cell infiltration. In streptozotocin (STZ)-induced diabetic mice, RECs can release cellular communication network factor 1 (CCN1) to induce neutrophil stasis and neutrophil extracellular traps (NETs) formation and extrusion [54]. Neutrophils and NETs are known to damage glycocalyx and increase vascular permeability [55]. Following clinical validation of its specificity and sensitivity, the secreted protein CCN1 could represent a valuable biomarker.

DR progression may also involve reduced HA synthesis, complementing HS degradation. Although HA constitutes a smaller fraction of glycocalyx, the loss can’t be neglected. Glycocalyx HA is a prerequisite for microvascular stability, acting both through its mechanosensitive properties and as a docking molecule for specific vascular stability factors, such as Ang-1 [56]. Loss of HA implies the disruption of Ang-1/Tie-2 signaling and following vascular destabilization [57, 58]. The early decrease of HA could be the effect of reprioritizations of glucose metabolism away from the HA biosynthetic pathways, although direct supporting data are currently lacking [59]. Interestingly, HA synthases seem to be activated by prolonged high glucose exposure, which is inconsistent with decreased HA levels [59]. This could suggest that hyaluronidases (enzymes that degrade HA) may play a more significant role. In diabetic ECs, up-regulated early growth response 1 (EGR1), a master transcription factor that coordinates EC activation, has been reported to bind to the promoter of hyaluronidase genes [56]. However, as EGR1 is not typically secreted extracellularly by ECs, its value as biomarkers remains uncertain.

#### 2.2.4 Loss of tight junction proteins

Contiguous expression of TJ proteins plays an important role in inner BRB (iBRB) integrity, restricting paracellular flux between cells [60]. TJs are mainly composed of transmembrane proteins like junctional adhesion molecules (JAMs), occludins and claudins [61]. Their organization and formation of TJs are governed by peripheral proteins, including zonula occludens (ZO)-1 to -3, which act through multiple protein-protein interaction domains [62].

Occludin was the first TJ protein identified. Interestingly, it may not be required for intact TJ formation. Instead, evidence suggests a prominent role in the organization and stabilization of the TJ complex [60]. Overactivated glucose metabolism related hexosamine biosynthetic pathway induces the increase in protein O-GlcNAcylation [8]. This post-translational modification can affect various endothelial proteins, competitively modifying proteins expressed in the endothelium, such as Connexin43 (Cx43) to reduce ZO-1 and occludin [63]. Besides direct effects of high glucose stress, indirect processes must be considered. Tie-2 shedding following the pericyte loss has been reported [37], and subsequently reduced production and effects of serum proteins, such as leukocyte cell-derived chemotaxin 2 (LECT2) worsen the loss of ZO-1 and occludin [64]. Serum LECT2 levels have been reported to be negatively correlated with the presence of DR in patients [65]. However, as LECT2 is primarily produced by hepatocytes [66], its specificity as a direct indicator of retinal vascular changes requires further investigation.

Additionally, at the iBRB, claudin-5 is the most highly expressed claudin protein and a key TJ component [67]. Thus, deficiency of claudin-5 likely has a significant impact on barrier function. Attenuated expression of claudin-5 in HRMEC treated with high glucose has been correlated with decreased levels of C1q/TNF-related protein-3 (CTRP3), an adiponectin paralog [68]. Serum CTRP3 levels have been shown to differ between patients with and without DR [69]. However, whether the changes of serum CTRP3 levels precede visible vasculopathy still needs further exploration.

#### 2.2.5 REC and reactive gliosis

Although vascular and neuronal lesions clearly both occur during DR, the casual order of vasculopathy and neuropathy remains debated [5]. Some studies observing impaired neuroretinal function (e.g., via electro-retinography (ERG) or optical coherence tomography (OCT)) without concurrent observable vascular abnormalities have led to the conclusion that neuropathy is prior to vasculopathy [5]. However, the lack of observable vascular defects can’t confirm the unaltered vessel function. Most factors released from stressed RECs can directly interact with, or indirectly affect neuronal cells, via astrocytes and microglia. Reactive gliosis is the hallmark of DR [70].

RECs interact with astrocytes via the gap junctions, such as Cx43 (mainly expressed in astrocytes) [71]. Excessive reactive oxygen species (ROS) can be transported from RECs to astrocytes through these gap junctions. ROS exposure can trigger downregulation of brain-derived neurotrophic factor (BDNF) expression in Müller cells (a unique type of retinal glial cell with astrocyte functions). Reduced BDNF levels initiated the neuroretinal dysfunction, detectable as reduced oscillatory potentials (OPs) amplitude on ERG [72]. OPs amplitude changes have been shown to be significantly different in NDR patients compared to healthy individuals [73]. Additionally, inflammatory factors associated with DR, such as IL-1β, TNFα, IL-8, and IL-6, have been verified to propagate between RECs and Müller cells [74], contributing to the inflammatory milieu.

In addition to interacting with astrocytes, RECs can release factors to stimulate microglia activation, thereby indirectly affecting neuronal cells. Under high glucose conditions, RECs release colony-stimulating factor 1 (CSF1), which interacts with CSF1 receptor on microglia to stimulate activation and inflammatory factors release [75]. Activated microglia have been proven to increase contact with and engulfment of amacrine cells and synapses [6]. Notably, activated Müller cells can amplify the neuroinflammatory effect mediated by microglia [76].

#### 2.2.6 Retinal endothelial-to-mesenchymal transition

Endothelial-to-mesenchymal transition (EndoMT) is an epigenetically regulated process wherein RECs lose endothelial characteristics and aquire mesenchymal-like phenotypes. This process contributes to early endothelial dysfunction in DR. In the early stage, prevented EndoMT (e.g., through miR-9 overexpression) has been verified protective roles on the BRB [77], suggesting that RNA-based biomarkers related to EndoMT might hold screening value.

Furthermore, EndoMT may be induced by lactate overload in RECs. It has been experimentally proven that excessive lactate stimulates the occurrence of EndoMT in other biological contexts [78, 79]. For instance, in ischemic myocardial tissue, excess lactate in the ECs promotes the expression of Snail1, an EndoMT-promoting transcription factors [78]. In ischemic skin flaps, excess lactate can promote the nuclear translocation of Twist1, another key EndoMT transcriptional regulator [79]. However, the potential link between lactate and EndoMT specifically in the diabetic retina requires further investigation. While plausible based on elevated retinal lactate in DR and findings in other systems, direct evidence supporting this specific mechanism in RECs during DR is currently limited.

#### 2.2.7 REC senescence and death

Typically, cellular senescence and apoptosis are mutually exclusive cell fates. However, they may coexist in various stages of DR. REC senescence and apoptosis are devastating for iBRB.

Cellular senescence is the state of stable cell cycle arrest and antagonizes cell proliferation, including two main types of senescence: replicative and premature [80]. The presence of REC senescence in DR and its role in promoting disease progression have been widely demonstrated [52, 81–85]. Retinal pathological angiogenesis in PDR patients and mouse models of OIR is associated with premature REC senescence [81, 83, 84], while replicative senescence is associated with microvascular abnormalities in NPDR patients. But interestingly, the senescence-related molecules in aqueous humor and vitreous humor from NDR and NPDR patients are not significantly different from those of normal people [85]. The observation might suggest that REC senescence is less common, or its secretory phenotype is less pronounced in the early stage of DR. Susceptible RECs develop replicative senescence under high glucose stress and exhibit senescent phenotypes. This aligns with the findings by Crespo-Garcia et al., who identified a REC cluster with the senescent phenotype in the retina of STZ-induced diabetic mice [82]. Hyperglycemia-induced activation of the cyclic GMP-AMP synthase (cGAS)/stimulator of interferon genes (STING) pathway has been proposed as a mechanism driving REC senescence in early DR [52]. Downstream signaling may involve increased p53 activity, which can promote the ubiquitination and degradation of Forkhead box O3 (FoxO3a) to aggravate REC senescence [86].

RECs exhibit a distinct metabolic property characterized by high rates of aerobic glycolysis, irrespective of oxygen availability [87]. This metabolic preference leads to substantial intracellular lactate accumulation following excess glucose influx [10], thereby providing sufficient substrates for lactylation. Histone lactylation is a brand-new epigenetic modification relying on lactate produced by intracellular metabolism and it regulates cell biological functions by activating downstream gene transcription and expression [88]. The role of histone lactylation has begun to be explored in DR. Chen et al. reported that hyperglycemia-induced lactate elevation promotes histone lactylation within the promoter region of the FTO gene. The resulting increase in FTO protein levels can induce mRNA demethylation, affecting the expression of downstream targets and ultimately contributing to microvascular abnormalities [40]. Fan et al. reported that VEGF-induced lactate promotes elevated H3K9la levels at angiogenesis-related genes in RECs [89]. However, lactylation-induced REC senescence has not been reported so far. In contrast, the relationships between histone lactylation and cellular senescence have been extensively studied in other pathological conditions and cells, including vascular smooth muscle cells in atherosclerosis, microglia in Alzheimer's disease, renal tubular epithelial cells in diabetic nephropathy, human nucleus pulposus cells in intervertebral disc degeneration, and tumor cells in lung adenocarcinoma [90–94]. These findings raise the question of whether similar mechanisms operate in RECs, although this remains speculative. A potential mechanism could involve increased histone lactylation at the promoter regions of genes encoding components of SASP, thereby promoting their expression. SASP refers to the complex secretome of senescent cells, comprising various bioactive molecules like IL-1β, IL-6, and TNF-α [84, 90]. The detrimental role of REC SASP in promoting pathological angiogenesis and impairing iBRB integrity in DR has been validated [81, 83, 84, 95]. Although there has been limited studies and direct evidence on lactylation-induced REC senescence to data, it represents a plausible working hypothesis and an innovative area for future research. Notably, the reason why few people pay attention to REC senescence in the early stage of DR is that the fact that significant SASP differences are difficult to observe in the early stages of DR due to lower prevalence of REC senescence. Better understandings of this process help to identify and intervene early.

Acellular capillaries are typical pathological features of NPDR, which are caused by the loss of pericytes and RECs. Retinal capillary cells undergo accelerated death, which precedes the development of characteristic histopathologic lesions in DR [96, 97]. Apoptosis and pyroptosis are the principal modes of REC death in this context.

Increased peroxynitrite levels have been shown to induce REC apoptosis under high glucose conditions *in vitro* and in diabetic animal models *in vivo* [98, 99]. Increased ROS and dysregulated NOS induce preoxynitrite formation. Peroxynitrite can disable p85, a regulatory subunit of PI3K, by tyrosine nitration and subsequently induce apoptosis by activation of proapoptotic p38 MAPK signaling pathway and inhibition of the pro-survival Akt signal in RECs [98]. Furthermore, NO, in conjunction with elevated homocysteine levels also observed in diabetes, can exacerbate mitochondrial dysfunction by increasing its nitrosylation of mitochondrial fission proteins under high glucose conditions [100].

Cx43 is an important gap junction component in RECs. High glucose can activate RhoA/ROCK axis to induce Cx43 internalisation [101]. This may impair the intercellular transfer of molecules, potentially including antioxidants or detrimental species like superoxide, thereby exacerbating REC apoptosis. Moreover, Cx43 exerts non-canonical functions within EC mitochondria, influencing apoptosis regulation [102]. Hyperglycemia-induced Cx43 deficiency is associated with mitochondrial fission and morphological abnormalities [103]. Since cytoplasmic Cx43 upregulation can lead to increased mitochondrial Cx43 localization [102], hyperglycemia might affect mitochondrial fission and subsequent apoptosis by reducing overall Cx43 expression, altering its translocation, and promoting its degradation.

Many miRNAs are differentially expressed in normal and diabetic retinal tissues, and their aberrant expressions often correlate with the progression of DR [104]. Studies highlight critical roles of miRNA in cellular death in DR. High glucose induces decreased miR-145 levels in RECs. Its direct target toll-like receptor 4 (TLR4), plays an important role in REC apoptosis. Overactivation of TLR4 promotes NF-κB signaling and the formation of pro-inflammatory factors, further amplifying local inflammation and inducing or exacerbating REC apoptosis [105, 106]. In DR patients, up-regulated miR-29b-3p can promote REC apoptosis by reduced Sirt1, as Sirt1 has been identified as the direct target of miR-29b-3p [107]. The mechanism by which reduced Sirt1 contributes to apoptosis in this context remains unclear, but some investigators proposed that reduced Sirt1 induces p66Shc expression by increasing acetylation of its promoter area. p66Shc is a 66 kDa proto-oncogene Src homologous-collagen (Shc) homolog adaptor protein, which is considered as a sensor of oxidative stress-induced apoptosis [108]. p66Shc can indirectly promote NOX2 and directly oxidize cytochrome c (Cyt c) to generate ROS and induce apoptosis [109]. Cyt c release from mitochondria is a key apoptotic signal [110]. Hyperglycemia-induced excessive mitochondria fission is linked to Cyt c-mediated apoptosis, though mitochondrial fission is important in segregating the damaged mitochondria for degradation [111]. Mitochondrial fission proteins include dynamin-related protein 1 (Drp1) and fission 1 protein (Fig. 1) [112], whereas fusion is mediated by optic atrophy gene 1 (OPA1) (inner mitochondrial membrane) and mitofusins 1 and 2 (MFN1/2) (outer mitochondrial membrane) [113]. OPA1 acts as a gatekeeper regulating Cyt c release during apoptosis [114]. Under high glucose, OPA1 levels are deficient in RECs, leading to abnormal mitochondrial functions and morphology and cellular apoptosis induced by increased release of Cyt c [113]. Concurrently, the fission proteins Drp1 and Fis1 increase to make REC apoptosis significant [112, 115]. While the proposed roles for these microRNAs in REC apoptosis are compelling, further experimental validation specifically within the retinal context is warranted.

Separate from mitochondria-dependent apoptosis, Dorweiler et al. reported that the formation of ceramide-rich platforms is related to REC apoptosis. This process was induced by TNF-α and IL-1β in a dose- and time-dependent manner in bovine RECs [116]. The ceramide-rich platforms may serve as rheostats, transmitting the extent of membrane injury to the cell interior, ultimately leading to REC apoptosis [117]. Interestingly, they noted that high glucose can not induce ceramide-rich platforms formation or significant REC apoptosis within their experiment timeframe.

Apart from apoptosis, cellular pyroptosis has emerged as another important cellular death pathway in DR. Pyroptosis is an inflammatory form of programmed cell death executed by the pore-forming protein gasdermin (GSDM) family proteins [118–120], including GSDMD and GSDME. In RECs exposed to high glucose, downregulated *miR-590-3p* contributes to REC pyroptosis via a positive feedback loop [121]. High glucose makes *IL-1β* up-regulated. Increased IL-1β attenuates *miR-590-3p* expression by decreasing the DNA promoter activity. Downregulated *miR-590-3p* specifically induces the expression of *NADPH oxidase 4 (NOX4)* and *nod-like receptor family pyrin domain containing 1 (NLRP1)*. NOX4 is not only a ROS-producing enzyme, but also together with NLRP1 induces caspase-1, which is critical for activation of IL-1β and pyroptosis [121].

In patients with DR, elevated circulating levels of lipopolysaccharide (LPS) may synergize with high glucose. Combination of high glucose and LPS induces the large P2X7-associated pores and activation of NLRP3 to mediate atypical pyroptosis and apoptosis [122].

Interestingly, RECs also possess endogenous protective mechanisms against pyroptosis under high glucose conditions. Gasdermin proteins have been determined to trigger pyroptosis by glucose-induced activated caspase-3 cleavage. However, RECs upregulate tumor necrosis factor superfamily member 15 (TNFSF15) in response to hyperglycemia. TNFSF15 directly interacts with the pyroptosis-related protein GSDME to exert anti-pyroptosis effects and thus acting as an endogenous brake on this cell death pathway [123].

## 3. Early identification

The absence of screenable indicators capable of identifying earlier DR pathology before the appearance of visible vascular abnormalities hinders timely medical intervention. Advances in screening for NDR with dysfunctional retinopathy fall into three categories: information capture (what to use), analysis (what to determinate), and assessment (how to assess). Potential approaches include analyzing biomarkers from body fluids, refining the interpretation of parameters derived from existing diagnostic instruments, and leveraging cutting-edge technologies (Table 1).

Patients with type 1 and type 2 diabetes are at an increased risk for developing DR. Regular screening with comprehensive eye examinations is recommended for these individuals, as symptoms often do not manifest until the disease has advanced and vision is threatened [124, 125]. Unfortunately, screening adherence remains suboptimal, with estimates suggesting that less than 50% of patients with diabetes receive appropriate ophthalmic evaluations following referrals from primary care physicians [126, 127]. To facilitate earlier detection of retinopathy in a larger proportion of diabetic patients, more sensitive and specific screening indicators are urgently needed. A prerequisite for the selection of screening indicators is their ability to demonstrate significant differences between NDR patients (those with subclinical functional deficits) and healthy individuals, irrespective of whether these NDR patients already differ significantly from those with established NPDR. This section reviews studies published within approximately the last 3-5 years focusing on individuals classified as NDR.

### 3.1 Valuable parameters

#### 3.1.1 Assessing neural damage

Current DR grading systems recognize the funduscopic detection of microaneurysms as the first visible sign [128]. However, retinal microvascular and functional changes often precede that and can potentially be detected through evaluations of color vision, contrast sensitivity, and ERG [129–132].

Non-invasive fundus imaging and electrophysiology are commonly employed in clinical practice due to their convenience, patients’ acceptance and relative affordability. Therefore, identifying valuable parameters within these existing modalities to evaluate early retinopathy in NDR patients represents a feasible approach.

OCT is a non-invasive imaging technique that provides cross-sectional views of the retina. In the context of neuroinflammation, OCT detection of hyperreflective foci (often described as hyperreflective dots or HRD), potentially representing activated microglia or macrophages near the vitreoretinal interface, may hold predictive value, as such cells are sparse under physiological conditions [133]. Spectral domain OCT (SD-OCT) is frequently used for this purpose. It has been reported that these HRD in the diabetic retina, delineated on SD-OCT, likely represent activated glial cells, and their number tends to increase with retinopathy progression [134]. HRD may present in the diabetic eyes when clinical retinopathy can’t be detected [135], suggesting their potential utility for closely monitoring DR risk in clinical practice. Incorporating artificial intelligence (AI) appears promising for automating the identification and quantification of HRD [136]. Some investigators have demonstrated high sensitivity and specificity (around 95%) for AI algorithms identifying diabetic macular edema based on hyperreflective points and hard exudation [137]. However, specific and feasible protocols for using HRD as an early DR biomarker are currently lacking. Sensitivity, specificity, typical distribution patterns, and quantitative cut-off values require determination through large-scale clinical studies. Additionally, the reduction of outer retinal reflectivity may be a potential biomarker of early retinal alterations and outer retinal reflectivity measurement is also suggested for assessing retinal nerve damage, particularly in patients with long-standing diabetes or poor glycemic control [138]. Recently, the LIFE-Adult-Study demonstrated that impaired glucose homeostasis is associated with thinning of the optical bands of retinal outer nuclear layer and photoreceptor myoid zone [139]. Given that impaired glucose tolerance reflects underlying insulin resistance, SD-OCT-detected thinning in these layers may serve as a valuable predictor of early retinal changes.

**Table 1.** Summary of parameters for early detection.

Parameters	Method	Subjects	Ref.
**Quantities of macrophage-like cells proximity to the vitreoretinal interface**	OCT	Mice	[133]
**Quantities of hyperreflective intraretinal dots**	SD-OCT	Normal, NDR, DR	[134]
**Thickness of outer retinal reflectivity**	OCT	Normal, NDR	[138]
**Thickness of the optical bands of retinal outer nuclear layer and photoreceptor myoid zone**	SD-OCT	Normal, prediabetes, diabetes	[139]
**The implicit time of DA 10.0 and OP2, OP 1-3 amplitudes**	ffERG	Normal, NDR, DR	[73]
**The R2 amplitude, the R4 and R5 implicit times**	mfERG		
**Perfusion density of macular and optic nerve head**	OCTA	Normal, NDR, DR	[129, 143–145]
**The foveal avascular zone areas and perimeters**			
**Vascular density**		Diabetes, NDR	[146]
**Intraocular and interocular vascular density variance**		NDR, DR	[147]
**The choroidal capillary perfusion**	SD-OCTA	NDR, DR	[155]
		Mice	[156]
**Adhesive fluorescent nanoprobe**	live retinal microscopy	Mice	[200]
**Cortistatin**	Aqueous humor	Normal, NDR, DR	[159]
**VEGF_165_b/VEGF ratio**		Normal, NDR, DR	[162]
**NCAM1, NRXN, and SPARCL1**		Normal, NDR	[163]
**miRs (-146a, -21, and -34a)**	Serum	NDR, DR	[171]
**miR-122**		Normal, NDR, DR	[174]
**Lymphocyte-to-monocyte ratio, neutrophil-to-lymphocyte ratio and platelet-to-lymphocyte ratio**		NDR, DR	[175]
**Down-regulated proteins: three ceramides and seven sphingomyelins, up-regulated protein: one phosphatidyl-choline, two lysophosphatidylcholines and two sphingomyelins**		NDR, DR	[180]
**FABP4**	Serum	NDR, DR	[181]
**Gut microbiome**	Fecal sample	Normal, NDR	[184]
**Heat shock protein 27, lipocalin1, beta-2 macroglobulin**	Tear	Normal, NDR, DR	[188, 189]
**Tear lipocalin 1, lactotransferrin, lacritin, lysozyme C, lipophilin A and Ig lambda chain C region**		Normal, DR	[190]

Full-field ERG (ffERG) and multifocal ERG (mfERG) are non-invasive, objective and sensitive methods to detect subtle retinal changes in clinical trials [140], with the potential to identify NDR patients with dysfunctional retinopathy. Whereas ffERG records summed electrical response from the entire retina, mfERG can detect localized abnormalities across different regions of the retina [73]. International Society for Clinical Electrophysiology of Vision has developed standard ERG protocols to ensure comparability and reliability of results [141]. Under dark-adapted (DA) conditions, the ffERG includes responses to flash strengths of 0.01, 3 and 10 phot cd·s·m^-2^ (DA 0.01, DA 3 and DA 10) [141]. Subclinical scotopic ffERG biomarkers of DR include reduced rod-initiated function (b-wave amplitude reduction or implicit time prolongation) and alterations in amacrine-mediated OPs [73]. It has been demonstrated that the parameters such as the implicit time of DA 10.0, the OP1-3 amplitudes and OP2 implicit time in ffERG could significantly differentiate NDR patients with dysfunctional retinopathy and DR patients from non-diabetic controls [73]. Furthermore, because retinal lesions in diabetes are often unevenly distributed, mfERG may be particularly suited for detecting the earliest localized functional anomalies. Studies analyzing mfERG ring averages have reported that parameters such as reduced R2 amplitude and extended R4 and R5 implicit times can show differences between NDR patients with dysfunctional retinopathy and healthy subjects [73].

#### 3.1.2 Assessing retinal ischemia

Retinal vasculature parameters can reflect the changes of retinal vascular network for showing the development of DR and even cerebrovascular disease [142].

Optical coherence tomography angiography (OCTA) has recently emerged as a tool for evaluating fundus vascular status in NDR patients. OCTA can reveal early fundus alterations in perfusion density and the extent of non-perfusion areas, which are indicators of retinal ischemia for NDR patients with dysfunctional retinopathy [143, 144]. Compared to healthy eyes, eyes of NDR patients with dysfunction retinopathy have been shown to exhibit lower perfusion density in the macula and around the optic nerve head, along with larger FAZ areas and perimeters [129, 145]. Moreover, NDR patients with dysfunctional retinopathy have decreased VD [146] and greater intraocular and interocular VD variance, which may be a more sensitive indicator of early vascular change than VD itself [147]. However, the clinical value of VD variability requires further demonstrations, particularly with comparisons to healthy controls, which were lacking currently. Moreover, a longitudinal study reported a 14.4% rate of conversion to DR per year among NDR eyes and these eyes exhibited quicker reduction of parafoveal perfusion density in superficial capillary plexus [148]. OCTA can aid in patient education regarding imminent visual threats and the necessity of tighter glycemic control, especially since NDR patients with dysfunctional retinopathy may not perceive subtle visual impairments [149].

However, establishing a standardized OCTA screening protocol for NDR patients faces challenges. One issue is the high inter-individual variability of parameters like FAZ area, even in normal eyes. Although normative datasets are available for different commercial OCTA machines, factors such as ethnicity, gender, and age may confound these measurements [150, 151]. For example, gender is an independent factor influencing the FAZ size, with normal adult females typically having larger FAZ areas than males due to thinner fovea [147]. When developing protocols, monitoring temporal alterations is required. In addition, the non-perfusion areas in deep capillary plexus have been shown to colocalize with disruption of photoreceptors in the macula of DR eyes [152]. Some researchers found that delayed ERG implicit time in NDR patients with dysfunctional retinopathy correlated with reduced perfusion density in parafoveal superficial capillary plexus [130]. This highlights the potential value of integrating functional and vascular assessments, requiring further large-scale studies to establish robust combined screening criteria

In addition to the changes in superficial capillary layers perfusion and vascular density detected by OCTA, the choroidal capillary perfusion alterations detected by swept-source OCTA (SS-OCTA) have also been shown to predict the occurrence of retinopathy in NDR patients. Significantly reduced choriocapillaries were reported in NDR patients with dysfunctional retinopathy in comparison with nondiabetic individuals [153] and it’s positively associated with the DR severity [154]. A 3-year longitude cohort study provides recent evidence that reduced choriocapillaries perfusion in eyes with diabetes precedes retinal vascular changes [155]. Furthermore, in the diabetic mouse model, reduced choroidal perfusion was observed before retinal perfusion changes or visual function abnormalities [156]. The percentage of choriocapillaries flow defect has been found to be independently associated with the risk of 3-year DR progression, potentially suggesting it may be a more valuable predictor for DR beyond retinal vessels [155]. Although standardized quantitative parameters and thresholds for choriocapillaries flow defects are yet to be defined, this represents a promising avenue for future research.

In the initial phase of DR, retinal swelling due to damaged iBRB and deposition of lipoprotein secretions into retinal tissue induce exudates formation [157]. According to the progression of retinal microvascular pathological lesions, impaired vascular function should precede structural abnormalities, suggesting that exudates would be useful for screening NDR patients with dysfunctional retinopathy. However, the modified Airlie House classification show that hard and soft exudates typically appear after microaneurysms, which are considered the earliest visible vascular changes in DR [158]. This apparent discrepancy may arise because early, diffuse vascular leakage might not form distinct, visible exudates detectable by standard color fundus photography until significant accumulation occurs. This suggests limitations in the sensitivity of conventional color fundus photography for detecting the earliest functional vascular changes characteristic of the NDR stage.

### 3.2 Potential biomarkers

#### 3.2.1 Aqueous humor and vitreous biomarkers

Biomarkers from vitreous or aqueous humor may be sensitive enough to detect early intraocular changes. For instance, the detection of proteins expressed only in the liver can indicate compromised vascular barrier integrity [85]. While aqueous and vitreous humor samples offer high reliability for reflecting the intraocular environment, their invasive collection limits their widespread use in screening, resulting in fewer studies compared to less invasive sample types. Mehmet Balbaba et al. demonstrated that aqueous humor cortistatin level can distinguish NDR and NPDR patients from healthy normal individuals, while serum cortistatin levels did not differ significantly [159], which also provides evidence for the sensitivity of aqueous humor detection.

The utility of vascular endothelial growth factor (VEGF) as an early biomarker is debated. VEGF plays an important role in the progression of DR, and serum VEGF levels can distinguish NDR patients from healthy people and are positively correlated with DR severity [160, 161], suggesting its potential as a systemic biomarker. However, other studies have found that VEGF levels in the aqueous humor of NDR patients are not significantly different from those in healthy normal people [85, 162, 163]. Consequently, the VEGF165b/VEGF ratio in the aqueous humor has been proposed as a more sensitive indicator. Multivariate logistic regression analysis has shown this ratio to be an independent factor negatively associated with DR progression, suggesting predictive value [162].

Recently, tandem mass tag liquid chromatography-tandem mass spectrometry was employed to identify differentially expressed proteins in the aqueous humor of NDR patients compared to healthy controls. Subsequent bioinformatic analysis identified potential novel mediators of neuronal dysfunction in early DR, including Neural Cell Adhesion Molecule 1 (NCAM1), Neurexin, and SPARCL1, whose differential expression was subsequently validated by western blot [163]. Among these, NCAM1 has the strongest established link to DR. In diabetic animal models, decreased levels of polysialylated NCAM have been associated with retinal ganglion cell degeneration [164, 165]. NRXN has been shown to regulate synaptic assembly and neurotransmitter release in the brain [166, 167], while direct evidence of the association of NRXN with DR is lacking. SPARCL1 has been identified as a secreted protein that can induce synaptogenesis in retinal ganglion cells [168]. This study also provides molecular clues supporting the hypothesis that retinal neurological impairment may precede vascular structural changes. In addition, in a gender-based analysis of aqueous humor proteins, Zeeshan Haq and colleagues found significant positive correlations between male gender and the concentrations of 12 proteins in NDR patients, including chemokines, proteases, proteins involved in programmed cell death, and a T-cell surface protein [169]. This underscores the necessity of considering potential sex differences when developing screening protocols based on aqueous humor protein concentrations.

#### 3.2.2 Blood biomarkers

Although the biomarkers of aqueous humor and vitreous humor are highly sensitive and specific for the detection of NDR patients with dysfunctional retinopathy, the patient acceptance of these sampling methods is low. In contrast, less invasive samples like blood, tears, or stool are generally more acceptable to patients for screening purposes. Among these, blood-based biomarkers have garnered significant research attention.

Circulating microRNA (miRNA) expression profiles have been reported as a potential biomarker for disease detection for various diseases [170]. Some investigators reported that serum miRNAs levels (e.g., miR-146a, miR-21, and miR-34a) show altered expression patterns along with DR progression. miR-146a is particularly noteworthy. miR-146a expression appears sensitive enough to differentiate between mild NPDR and NDR patients [171]. However, robust data that the serum miR-146a of NDR patients is statistically different from that of normal people can demonstrate its screening potential, which is still lacking. Notably, the alteration of miR-146a in DR seems conflicting. In patients with type 2 diabetes, serum miR-146a levels increased with the severity of DR [171]. Conversely, in the cohort of patients with type 1 diabetes, serum miR-146a levels were inversely correlated with DR risk [172]. The reason for this discrepancy related to diabetes type remains unclear. However, However, serum miR-146a levels appear unaffected by age or sex [172], and that polymorphisms in the gene encoding miR-146a do not affect DR susceptibility [173], so miR-146a may be a reliable biomarker candidate. Serum miR-122 levels have also been shown to predict NDR patients with dysfunctional retinopathy. Compared to healthy controls, miR-122 levels are higher in NDR and NPDR patients but lower in PDR patients. This discrepancy may be due to the biological function of miR-122 in anti-angiogenesis [174].

Some researchers supposed that systemic inflammatory cell ratios, such as lymphocyte-to-monocyte ratio (LMR), neutrophil-to-lymphocyte ratio (NLR) and platelet-to-lymphocyte ratio (PLR) can be cost-effective indicators of underlying inflammation in patients with NDR [175]. However, the value of these indicators is controversial. Some studies report their limited predictive value [176], while others consider them to be good predictors for distinguishing NDR patients with dysfunctional retinopathy [177–179]. Systemic inflammatory markers are easily influenced by various concurrent conditions, potentially limiting their specificity and sensitivity for DR screening. Establishing reliable cut-off values is also challenging, and in conjunction with other indicators rather than as standalone screening tools may be helpful.

Recent studies have begun to explore serum lipidomic profiles. One study using serum from patients with NDR and DR identified significantly different lipid molecules (down-regulated proteins: three ceramides and seven sphingomyelins; up-regulated protein: one phosphatidylcholine, two lysophosphatidylcholines and two sphingomyelins), proposing them as potential serological markers for DR presence in type 2 diabetic patients [180]. However, the study lacked healthy controls and could not ascertain the presence of subclinical retinopathy in NDR patients, making it difficult to determine the relevance of these specific lipid changes for early screening. Nevertheless, given the proposed role of ceramides (e.g., C16-ceramide) in REC apoptosis and DR progression [116], lipid molecules remain markers of interest, although further validation of their specificity and sensitivity in serum, particularly in well-defined NDR cohorts versus healthy controls, is required.

Additionally, fatty acid-binding protein 4 (FABP4), a known predictor for early diabetic nephropathy, has been investigated as a potential predictor for DR [181]. While average serum FABP4 values showed differential trends across DR severity groups in that study, direct comparison data between healthy controls and NDR patients was lacking.

#### 3.2.3 Fecal microbiome biomarkers

In addition to the most common blood samples, fecal samples have also gained attention, driven by the concept of the "gut–retina axis". This hypothesis posits that the gut microbiome, modulated by diet, probiotics, or antibiotics, can influence the development of retinal diseases, and its significance as a potential modulator of eye diseases has been increasingly recognized [182, 183]. Yinhua Huang et al. proposed that stool microbiota analysis is a potential screening tool for retinopathy in patients with NDR [184]. At the genus level, decreased relative abundance of *Faecalibacterium*, *Eubacterium_hallii_group* and *Clostridium* genera, alongside increased *Blautia*, *Bifidobacterium* and *Lactobacillus* were observed in both NDR and DR groups compared to healthy controls [184]. It is noteworthy that Hao Wu et al. proposed that metformin treatment, common among diabetic patients, affects intestinal *Bifidobacterium* levels, contributing to improving glucose tolerance and enhancing the antidiabetic effects [185]. This raises questions about the specific value of *Bifidobacterium* as an independent screening marker for retinopathy in metformin-treated patients, highlighting the need to account for medication effects in microbiome studies.

#### 3.2.4 Tear biomarkers

Recently it has been reported that the impaired blood flow observed in DR can modulate the composition of tear fluid, suggesting that tears can reflect retinal changes despite the lack of direct anatomical connection to the retina [186, 187]. Early studies in 2012 used two-dimensional gel electrophoresis analysis to compare tear proteomes from NDR, NPDR and healthy controls and screen out some potential biomarkers (2 up-regulated proteins, 18 down-regulated proteins) [188], including heat shock protein 27, lipocalin1, beta-2 macroglobulin, whose association with DR has been verified [189]. Another 2012 study identified several tear biomarkers, including tear lipocalin 1, lactotransferrin, lacritin, lysozyme C, lipophilin A and Ig lambda chain C region [190]. However, studies did not include a direct NDR versus healthy control comparison or assess for subclinical functional changes in the NDR group. More recently, a proteomic analysis comparing tears from NDR patients to healthy controls identified hemoglobin subunit beta as being significantly up-regulated in NDR tears, with levels also elevated in PDR patients [191].

#### 3.2.5 Limitations

Although significant progress has been made in the exploration of biomarkers, limitations remain. On the one hand, selecting the optimal biomarkers is challenging. Systemic biomarkers in blood or urine are readily accessible, but may lack specificity for DR, being greatly affected by systemic conditions. Ocular biomarkers, whether from fluid samples (aqueous humor/vitreous humor) or based on imaging/electrophysiology, are more direct reflections of ocular status, but vary considerably in invasiveness, cost, technical requirements, and standardization. The most effective single biomarker or combination for widespread, cost-effective screening remains to be determined.

On the other hand, patient heterogeneity (e.g., diabetes type, duration, glycemic control, comorbidities, medications, genetics, sex) presents a significant challenge in identifying universally applicable biomarkers. Many proposed biomarkers demonstrate disease association rather than proven causality, limiting their immediate utility as precise predictors or therapeutic targets. Establishing the clinical utility and translational potential of any biomarker requires large-scale, prospective, longitudinal studies to validate predictive accuracy and assess real-world performance. Such studies are inherently costly, time-consuming, and susceptible to participant attrition, and promising findings from smaller, cross-sectional studies may not always be confirmed in larger validation cohorts.

### 3.3 Cutting-edge technology

#### 3.3.1 Adaptive optics retinal imaging

Adaptive optics scanning laser ophthalmoscopy (AO-SLO) represents a significant advancement for researchers and clinicians, providing high-resolution visualization of retinal microstructures in vivo at a cellular level [192, 193]. Previous studies have shown that AO-SLO can directly monitor the erythrocyte aggregates in retinal capillaries and spatiotemporal blood flow images are used to visualize blood corpuscle trajectory [194]. Based on the analysis of erythrocyte aggregate alongation rates from spatiotemporal blood flow images, both NDR and NPDR patients show significantly reduced rates compared to healthy subjects, potentially indicating altered microvascular hemodynamics [194]. In addition, the wall-to-lumen ratio (WLR) detected by AO-SLO, has been investigated as the most accurate marker of retinal vascular remodeling. Significant differences of WLR can be observed between NDR and healthy subjects [195]. However, multiple linear regression showed that hypertension has strong effects on WLR of NDR patients, confounding its interpretation as a specific marker for early DR-related vascular changes [196]. To address such confounders, a novel metric, the arteriole index ratio (AIR), has been proposed. This index quantifies structural parameters of individual vessels relative to a normative young, healthy control population, allowing for averaging across vessel segments within an individual [197]. Compared to controls, patients with NDR exhibited a trend towards decreasing AIR, similar to that observed in NPDR patients, although the difference for the NDR group did not reach statistical significance [198]. More recently, AO-SLO has been utilized to quantify the density of retinal arteriolar vascular mural cells (VMCs). Studies have reported lower arteriolar VMC density in NDR subjects compared to healthy controls. Notably, the potential concern that age or hypertension could be confounding variables for arteriolar VMC density analysis has been addressed [199].

#### 3.3.2 Nanoprobe-based detection

One research group developed a high-brightness, adhesive fluorescent nanoprobe using biodegradable materials. The nanoprobe selectively targeted the VEGF receptor 2 (VEGFR-2), which is up-regulated in diabetes, even under dynamic flow conditions. After the systemic injection in diabetic mouse models, the nanoprobes adhered in the retinal microvessels and were visualized as bright spots in live retinal microscopy [200]. These functionalized and biocompatible nanoprobes represent a potential novel approach for diagnosis of NDR with high specificity and quantitative accuracy. However, the cost of this nanomaterial could be a barrier, particularly given existing difficulties with patient adherence to even standard, less expensive screening methods. Furthermore, significant research, including establishing diagnostic thresholds and demonstrating safety and efficacy in humans, is required before clinical translation can be considered.

#### 3.3.3 Multi-modal imaging

Recently, multi-modal imaging approaches combining measurements of oxygen saturation and blood flow have been explored to estimate inner retinal oxygen delivery and metabolism. Although the target index showed no significant difference between NDR patients and healthy normal people, and the differences were primarily observed in patients with severe NPDR and PDR [201], which means that oxygen delivery in the early stage of retinopathy is not greatly affected, the assessment of retinal oxygen content by non-invasive means is a major improvement, and it is more suitable for tracking the progression of DR than NDR screening.

#### 3.3.4 Computational approaches and screening logistics

AI is attracting attention in the medical field. AI algorithms offer potential for efficiently screening large datasets to identify novel biomarkers or optimize the interpretation of existing parameters. A team has used different AI algorithms to identify several immune-related molecules (e.g., FCGR2B, CSRP1, EDNRA, SDC2, TEK, and CIITA) as DR biomarkers [202]. These molecules are relatively understudied in the context of DR, and their value has only been validated in animal models [203, 204]. Among them, FCGR2B expression levels in NDR patients were significantly higher than those in normal people [205], but the practical value of FCGR2B and other factors selected by AI algorithms remains to be elucidated.

Beyond technological advances, addressing practical barriers to screening is crucial. Healthcare disparities exist, and individuals at the highest risk for DR often exhibit lower screening uptake rates [206]. Teleretina screening represents a viable strategy, involving digital transmission of ocular images acquired by a technician for remote evaluation by a specialist [207]. There are also teams that integrate data from clinical practice (four independent risk factors, including hypertension, blood urea nitrogen, duration of diabetes, and diabetic peripheral neuropathy) to construct the nomogram to help primary care physicians quickly identify individuals at high risk of developing DR in patients with type 2 diabetes [208].

## 4. Promising treatment strategies

A screening program alone is insufficient to reduce vision loss and timely referral for ophthalmological interventions is essential [206]. Current treatments for DR, such as intravitreal anti-VEGF drugs and retinal laser photocoagulation, primarily target pathological neovascularization, the hallmark of PDR, aiming to impede disease progression [209]. A critical gap exists in effective early intervention strategies, potentially missing opportunities to halt or reverse pathology. Early intervention encompasses both patient-self management and medical intervention.

### 4.1 Patient-self management

A South Korean study showed the prevalence of comorbidities, finding that 53.2%, 61.3%, and 72% of diabetic patients also had obesity, hypertension, and hypercholesterolemia, respectively. Among these subjects with diabetes, 43.7% had both hypertension and hypercholesterolemia. With regard to glycemic control, only 28.3% reach the target levels. A mere 11.5% of them meet targets for glycosylated hemoglobin, blood pressure, and lipids concurrently [210]. Patients with prolonged high glucose even develop worse outcomes after cerebrovascular diseases [211]. A recent large cohort study demonstrated multiple healthy lifestyle behaviors, including a low waist circumference, noncurrent smoking, ideal habitual diet, regular physical activity, and moderate alcohol intake could jointly affect retinopathy [212]. Participants adhering to 4-5 low-risk lifestyle behaviors had a 35% lower risk of DR compared to those adhering to 0-1, with each additional low-risk lifestyle behavior associated with a 13% lower risk [212].

For those who have achieved very low glycemic targets since diagnosis, the risk of vision-threatening DR is greatly reduced [213], with a threshold below which they may not develop DR until 24 years’ duration of type 1 diabetes [214]. However, although intensive glycemic control could significantly reduce the incidence of DR to less than 50% [215, 216], the remaining patients are still at high risk of DR [209, 217]. A recent study provided an insight that this population may exhibit lower serum ethanolamine levels, compared to those without retinopathy [218]. In animal models, ethanolamine supplementation can reduce DR-related inflammation and inhibit microglial diacylglycerol (DAG)-denpendent PKC pathway activation [218], which suggests that combinating hypoglycemic drugs with ethanolamine supplements can further slow the progression of retinopathy. In addition, “hyperglycemic memory”, where prior poor glycemic control continues to influence outcomes despite later improvement, may also contribute to DR progression in patients with currently well-controlled blood glucose. Animal models have demonstrated that insulin combined with systemic C-peptide supplementation can effectively alleviate retinopathy, potentially counteracting some aspects of this metabolic memory [219].

Dietary interventions are also relevant. The Mediterranean diet has shown benefits for DR, but recent studies have pointed to the mild toxicity of extra virgin olive oil, a staple of the Mediterranean diet, for ECs, supported by findings of reduced AKT activation and altered apoptosis-associated protein levels [220]. Additionally, nutraceuticals with antioxidant and anti-inflammatory properties may be used to treat retinal pathologies. Lisosan G, a fermented powder from whole grains, protects the retina from DR, while its efficacy was limited by poor bioavailability. Encapsulating Lisosan G within liposomes can greatly enhance its efficacy, achieving effects parallel to the highest dose of lisosan G [221]. It may pave the way for dietary supplements with improved therapeutic effects. In addition, the Mediterranean diet is rich in omega-3 fatty acids, but a recent study of the A Study of Cardiovascular Events iN Diabetes (ASCEND) reported that additional supplementation of omega-3 fatty acids has no significant clinical significance for the progression of DR [222].

#### 4.1.1 Traditional Chinese medicine

Traditional Chinese medicine (TCM) is a key therapy for preventing chronic diseases worldwide, and it has also received attention in the field of DR. A team used multiple important databases to screen out genipin with potential preventive effects. Genipin can decrease mitochondrial membrane potential and inhibit glucose uptake and energy metabolism in HRMECs exposed to high glucose to prevent from damage caused by glycosylation and phosphorylation [223]. However, these findings stemmed from experiments involving intraocular injection. Further research is necessary to determine if orally administered genipin, the typical clinical route for TCM, can achieve comparable protective effects within the eye.

Traditionally, Xiao Bopi is utilized to treat diabetic complications. Oral water extract of Xiao Bopi has been demonstrated protective roles for retinal ultrastructure and preventive roles for REC apoptosis by attenuating BAX/Bcl-2 ratio [224]. Jin-Gui-Shen-Qi Wan, another TCM formulation, has traditional therapeutic advantages in improving eye diseases. The study compared the therapeutic effects of metformin and Jin-Gui-Shen-Qi Wan on DR, and reasonable evidence showed that this TCM primarily protects retinal ganglion cells from apoptosis, with less pronounced effects on RECs compared to metformin [225]. A limitation noted was the lack of investigation into potential synergistic effects when combining metformin and Jin-Gui-Shen-Qi Wan, a common clinical scenario.

Ginseng, notoginseng and their polyherbal formulations have been widely used in the treatment of DR [226, 227]. Ginsenoside Rd is a representative triterpenoid saponin and is reported to ameliorate high glucose-induced REC apoptosis by AMPK-SIRT1 signal interdependence [228]. Recently, compound Danshen dripping pills (containing ginsenosides), were found effective in early-stage DR through vascular and neuroprotective mechanisms independent of glycemic control [229]. 20(R)-ginsenoside Rg3 inhibits endoplasmic reticulum stress, thereby alleviating RECs apoptosis and restoring the functional changes and retinal redox balance [230].

*Lonicerae Japonicae* Flos is a component of various anti-diabetic TCM formulas. Chlorogenic acid, as the main indicative compound of *Lonicerae Japonicae* Flos, was reported to be beneficial for DR [231]. Chlorogenic acid can reduce TNFɑ-induced EndoMT and leukocyte adhesion to RECs via directly interacting with TNFR1, thereby reversing iBRB breakdown [231, 232]. Qi-Ju-Di-Huang-Pill exerts anti-hypoglycemia, anti-inflammatory, anti-VEGF and anti-apoptotic effects, and can protect the retina, thus delaying DR progression [233]. The Heyingwuzi formulation protected RECs against apoptosis by mediating mitophagy *via* the HIF-1α/BNIP3/NIX pathway [234].

TCM may also complement conventional treatments. While essential, insulin therapy alone does not fully prevent DR incidence and paradoxically may carry some risk [235]. Insulin can directly activate Akt/mTOR signaling *in vivo* and induce HIF-1α/VEGF expression in RECs, causing vascular instability. Berberine, a compound found in several TCM herbs, can effectively control the progression of insulin-induced DR by inhibiting insulin-induced REC activation through the Akt/mTOR/HIF-1α/VEGF pathway [236].

#### 4.1.2 Antidiabetes agents

Beyond glycemic control, several antidiabetic agents exhibit direct protective effects on the retina. Dapagliflozin, a SGLT-2 inhibitor, can reduce apoptosis by decreasing arachidonic acid levels in RECs [237]. By reducing glucose uptake into RECs, it may mitigate intracellular glucose overload. ERG results of diabetic mice also showed that retinal nerve function was also improved with dapagliflozin treatment [238]. The above suggests that the utility of dapagliflozin is beneficial for patients with retinopathy. Other SGLT-2 inhibitors, such as empagliflozin, luseogliflozin and ipragliflozin, have also been shown to improve diabetic retinal damage [239, 240], where they particularly inhibit microglial activation and have a significant protective effect at low doses [240]. In addition, metformin, as a first-line drug for patients with type 2 diabetes, has been verified the therapeutic effect on DR by inhibiting oxidative stress-induced NF-kB/TLR4 pathways and suppressing glutamate excitotoxicity [241]. Linagliptin, a dipeptidyl peptidase-4 (DPP-4) inhibitor, has also been shown to protect RECs from TNFα-induced toxicity and enhance their viability [242]. Liraglutide, a glucagon-like peptide-1 (GLP-1) receptor agonist, reportedly exerts protective effects by restoring SIRT1 against senescent REC [243]. Moreover, metformin, SGLT-2 inhibitors, and alpha-glucosidase inhibitors also exhibit anti-aging effects independent of glycemic control [244]. Notably, although long-acting GLP-1 receptor agonist semaglutide exhibits neuroprotective effects in the cerebral ischemia-reperfusion injury models [245], it appears to be associated with increased risks of DR in individuals with type 2 diabetes also taking insulin [246].

#### 4.1.3 Lipid-lowering drugs

Lipid-lowering drugs commonly used by diabetic patients also have different effects on RECs. A systematic review of randomized controlled trials (RCTs) has proposed potential protective roles for statins [247], while Mendelian randomization and observational studies have revealed the causal relationship between DR risk and statins use [248]. Interestingly, the statins can mitigate the side effects of mesenchymal stem cell (MSC) therapy, which is a promising treatment strategy. Atorvastatin, one of the commonly used lipid-lowering drugs, may mitigate hypoxia-induced VEGF production from MSCs and enhance MSC viability and homing via the AMPK-eNOS pathway, potentially relevant for combination therapies [249]. Simvastatin protects against the early signs of DR by preventing NADPH oxidase-mediated activation of STAT3 [250] and exerts protective effects for retinal vessels in diabetic rats by inhibition of mitochondrial ROS/poly (ADP-ribose) polymerase (PARP) pathway mediated by proliferator-activated receptor gamma coactivator 1 alpha [251]. Fibrates, particularly fenofibrate, have garnered significant attention. Fenofibrate may serve as an adjunct therapy for ocular oxidative stress [252], improve EC survival via the AMPK pathway [253, 254], and inhibit metabolism memory of RECs through the Sirt1-dependent signaling pathways [255]. However, conflicting studies suggest limited effects on EC metabolic disorders, questioning its universal efficacy in preventing hyperglycemic complications [256].

#### 4.1.4 Antihypertensive drugs

Certain antihypertensive drugs may have direct retinal effects beyond blood pressure control. Captopril, an angiotensin-converting enzyme (ACE) inhibitor, has shown the reduction of glucose accumulation in the retina by inhibiting GLUT1-mediated glucose transport on RECs [257].

#### 4.1.5 Antiplatelet drug

Increased platelet aggregation, potentially due to reduced vascular prostacyclin production, contributes to diabetic complications [258]. Aspirin, besides reducing platelet aggregation, has been shown to significantly inhibit the increase in superoxide in the retina of diabetic animals [259] and reportedly inhibits abnormal microvascular formation and neuronal cell loss when administered orally [260]. Moreover, a large study of ASCEND provided reassurance regarding the ophthalmological safety of aspirin [261], supporting its potential utility in relevant patient populations.

Patient self-management also crucially involves adherence to recommended eye screening schedules. A recent parallel RCT suggested that targeted education alone is not enough, whereas autonomous AI-driven communication significantly improved eye examination completion rates among young people with diabetes [262].

### 4.2 Medical intervention

Beyond systemic control and lifestyle, specific medical interventions targeting the eye are under investigation, particularly for early stages.

#### 4.2.1 Non-invasive strategies

Non-invasive approaches are generally preferred by patients and can be divided into local and systemic strategies. Local prevention strategies often use eye drops, while systemic prevention strategies include oral medications, remote ischemic conditioning, intermittent hypoxia conditioning and so on.

Diabetes causes an imbalance of nerve growth factor (NGF) isoforms. The neuroprotective effect of local supplementation of NGF in the eye has been confirmed [263, 264]. NGF supplementation has been proposed as a secondary preventive strategy for DR, potentially initiated when early neuroretinal thinning (e.g., retinal nerve fiber layer/ganglion cell layer) is detected in NDR patients [265].

A group developed a noninvasive drug delivery system for efficient co-delivery of ellagic acid and oxygen by liposomes, which can be administered by eye drops. Liposomes are dually modified by two peptides that respectively mediate receptor recognition and internalization. Hemoglobin plays an oxygen-carrying role, reversibly binding oxygen and releasing oxygen where the partial pressure of oxygen is low [266]. Ellagic acid, an aldose reductase inhibitor, could remove excessive ROS to prevent retinal cell apoptosis [267] and promote retinal vascular normalization by regulating the VEGFR2 signaling pathway [268]. Significantly, after being administered, the liposome carrier obviously normalized the retinal vascular network and maintained the structural integrity of retina [266].

The topical administration of GLP-1 eye drops was verified to promote the release of γ-aminobutyric acid onto ganglion cells through the activation of GLP-1 receptor, leading to the de-excitation of ganglion cell circuits and the inhibition of excitotoxic processes associated with DR [269].

Administration of eye drops containing chitosan-modified 5-fluorouracil (5-FU) nanostructured lipid carriers (NLCs) can improve rat DR [270]. Although this kind of eye drops showed excellent preclinical efficacy, 5-FU is a chemotherapy agent with significant known severe side and toxic effects, such as fever, mucositis, leukopenia, neurologic abnormalities and so on [271]. The systemic side effects of 5-FU make its clinical application in non-oncological diseases questionable, despite of its strong anti-angiogenesis effect. However, chitosan-modified NLCs appear to be a promising delivery system. Chitosan and NLCs can provide more availability, residence time, improved permeation of effective medications to the targeted areas of the retina [270]. If this system can deliver all kinds of needed drugs, it would be the first choice.

Given the link between diabetes and cataracts, nanoparticles designed for topical co-delivery of pyrrolidine dithiocarbamate (PDTC) and triamcinolone acetonide (TA) have been developed, aiming for sequential release at the lens and retina. The combined utilization can achieve a moderate burst release of PDTC at the lens, followed by slow and sustained release of both PDTC and TA in the retina [272].

A major challenge for topical therapies is achieving therapeutic concentrations in the posterior segment due to low bioavailability and ocular barriers [273]. To address this, a team has developed drug-loaded bovine serum albumin nanoparticles coated with hyaluronic acid. Bovine serum albumin nanoparticles have been shown to permeate the cornea and have the potential to treat posterior segment diseases [274, 275], while the hygroscopic properties of hyaluronic acid allow it to form hydrogen bonds with mucins, so that it has values for targeted ocular delivery. There is no efficacy difference between eye drops and intravitreal injections. This indicates that the drug-carrying system has a high value for the early prevention of DR [276].

Oral medications related to NGF appear to be feasible. Imbalanced NGF isoforms can result in accumulation of its precursor proNGF and upregulation of the p75 neurotrophin receptor (p75^NTR^), with consequent activation of RhoA. *In vivo* studies reported that oral administration of LM11A-31, a small-molecule p75^NTR^ modulator and proNGF antagonist, significantly mitigates this accumulation and preserves iBRB integrity and *in vitro* experiment shows LM11A-31 can attenuate TJ protein loss in RECs [277].

Oral intake of eicosapentaenoic acid ethyl ester (EPA-E) can prevent retinal neurodegeneration in the early stage of DR. EPA-E is a type of dietary n-3 fatty acids and can contribute to the increase in an EPA metabolite, 18-hydroxyeicosapentaenoic acid (18-HEPE) in the eyes. Among metabolites in the retina after EPA administration, only 18-HEPE can induce BDNF upregulation in Müller cells and OPs recovery in ERG in DR [72]. Such dietary therapies should be more acceptable to patients

Limb remote ischemic conditioning (LRIC), involving cycles of thigh tourniquet inflation/deflation, showed protective effects in diabetic animal models. LRIC can play an anti-inflammatory and antioxidant role, and can also increase TJ protein expression, thereby improving DR [278, 279]. Although the protective mechanism of LRIC against DR is still unclear, it has been shown to play a protective role in mice with ischemic stroke by regulating ERK activity in peripheral blood and brain [280].

Intermittent hypoxia preconditioning (IHC) involves cyclic alternation between hypoxia and normoxia (hypoxia with 13% O_2_, hypoxic-normoxic intervals of 5 min for 10 cycles per day for 2 weeks) [281]. IHC has been demonstrated to protect against hypoxic-ischemic brain damage by promoting functional angiogenesis [281]. The protective effect of IHC on DR can be seen in our unpublished data. A disadvantage of systemic therapy is the potential for side effects in other systems.

#### 4.2.2 Invasive strategies

The most common route for delivering drugs to the posterior segment of the eye is intravitreal injection, which ensures the high local bioavailability.

##### 4.2.2.1 New uses for old drugs (new uses of conventional drugs or new indications for DR)

Nucleoside reverse transcriptase inhibitors (NRTIs) are mainstay therapeutics for HIV infection and recently its anti-inflammatory and anti-pyroptosis effects on DR have been verified [122, 282]. Lamivudine, one of the NRTIs, is proposed as a new P2X7 inhibitor and can attenuate neuronal and vascular defects in DR [283]. This conclusion is consistent with the experimental results of Hui Kong et al., who showed that lamivudine can inhibit the P2X7/NLRP3 inflammatory signaling pathway and the manifestations are similar to that observed after the specific inhibitors of P2X7 treatment [122]. However, concerns about known side effects, such as fatigue, gastroenterological symptoms, liver dysfunction and so on [284]. The benefits from NRTIs are latent and unproven, while the side effects are known and well documented. Until there is strong clinical evidence of their efficacy in DR, this balance strongly favors the risks over the potential benefits.

Systemic administration of insulin would greatly reduce the local medicinal properties of the drug. A group proposed a novel insulin delivering system via loading onto chitosan nanoparticles/poly (lactic-co-glycolic acid)-poly (ethylene glycol)-poly (lactic-co-glycolic acid) hydrogel (ICNPH). They demonstrated that a single subconjunctival injection of this insulin delivery system has sufficient neuroprotective effect on the retina of diabetic rat model and can also reduce the destruction of iBRB [285].

##### 4.2.2.2 Combination medications

Not all patients are sensitive to first-line anti-VEGF drugs. Some teams have proposed that inhibitors targeting the TNFSF15-GSDME axis combined with anti-VEGF drugs can produce cumulative or synergistic effects [123]. Anti-VEGF drugs such as conbercept can not only inhibit pathological angiogenesis, but also reduce REC pyroptosis, and TNFSF15 is a natural brake of GSDME-induced cell pyroptosis [123]. Testing the synergistic treatment potentials in preclinical DR models is warranted.

##### 4.2.2.3 Novel drug

Significantly reduced levels of the anti-inflammatory gaseous transmitter hydrogen sulfide (H_2_S) are observed in diabetic patients and correlate with microvascular dysfunction. H_2_S may protect microvasculature by preventing loss of endothelial glycocalyx. Diabetic animals received intraocular injection of the slow-release H_2_S donor NaGYY4137 or the mitochondrial-targeted H_2_S donor AP39 and the overall glycocalyx coverage and retinal vascular leakage improved [286]. If there is an oral H2S donor, it will be very clinically valuable.

Diabetic patients have damage to the vascular glycocalyx throughout the body, recent studies demonstrate that the glycocalyx can be therapeutically targeted with the novel heparanase inhibitor OVZ/HS-1638, a unique tetravalent dendrimer heparanase inhibitor with no-off target anticoagulant activity [287], to prevent microvascular dysfunction in diabetes in multiple vessel beds, such as eyes and kidney, in a mouse model of type 2 diabetes [288]. However, the drug is intervened by orbital injection. If this drug, which is suitable for acting on blood vessels throughout the body, can be developed, it will have great preventive potential.

Cell therapy has great potential to replace dead cells in the early stages of DR. *In vitro* studies have demonstrated MSCs can acquire endothelial-like markers and endothelial-like MSCs injected intravenously were able to restore altered vascular functions [288–290]. Additionally, endothelial colony-forming cells, a type of endothelial progenitor cell, can incorporate into pre-existing damaged capillaries, induce vasoprotective effects and prevent vision loss [291].

Cells-derived exosomes can also play a protective role. Bone marrow MSC-derived exosomes can attenuate glycocalyx degradation and vascular leakage via delivering miR let-7-5p to ECs [292]. M2 microglia-mediated exosomes normalize the EC/pericyte ratio, thereby protecting the BRB [293]. A limitation is lack of cellular specificity, which glycoengineering strategies aim to address [294].

A novel ω3-docosahexaenoic acid-derived lipid mediator, can enhance MSC functions, improving outcomes including reduced retinal pericyte loss in diabetic mice [295].

##### 4.2.2.4 New drug delivery systems

Recently, tetrahedral framework nucleic acids (tFNAs) have emerged as a hot drug delivery carrier [296]. A team has synthesized a delivery vector system tFNA-DJ-1-saRNA [297]. Protein deglycase DJ-1, an important endogenous antioxidant, is found to protect cells against oxidative stress-induced damage by ROS clearance and mitochondrial function protection [297]. Small activating RNAs (saRNA) are short double-stranded RNAs that can selectively promote the transcription of a gene by targeting its promoter region [298] and are facilized as a new technique to induce DJ-1 expression. These investigators found that tFNAs could penetrate the cell plasma membrane without any transfection agent, transfer DJ-1-saRNA into damaged ECs safely and efficiently, and significantly increase the expression of DJ-1 protein [297].

However, the acceptability of invasive treatments (like intravitreal injections) for prevention in asymptomatic or minimally symptomatic NDR/early NPDR patients is a significant consideration. Clinical decisions must carefully weigh the potential benefits against the risks, invasiveness, and patient burden, requiring clear evidence of necessity and effectiveness for such early interventions.

## 5. Conclusion and Future Directions

DR remains a leading cause of vision impairment globally, with early identification and intervention critical to mitigating irreversible damage. This review highlights the pathophysiological progression of DR at the RECs level, emphasizing the importance of recognizing "dysfunctional retinopathy" in patients classified as NDR. Key findings include the role of REC adaptive mechanisms (e.g., insulin resistance, low-grade inflammation) in early stages, followed by decompensation leading to BM thickening, pericyte and TJ proteins loss, glycocalyx degradation, EndoMT and REC apoptosis and senescence. Early biomarkers such as OCTA-derived perfusion metrics, ERG parameters, and serum miRNAs (e.g., miR-146a) show promise but require validation in large-scale studies. Emerging therapies, including SGLT-2 inhibitors, TCM (e.g., ginsenosides), and nanotechnology-based drug delivery systems, offer novel avenues for intervention but demand rigorous clinical evaluation.

Future directions should focus on:

1. Biomarker Development: Large-scale validation of candidate biomarkers (e.g., IGFBP7, CTRP3, choriocapillaris flow defects) and integration of multi-modal data (imaging, electrophysiology) to enhance sensitivity and specificity.
2. Precision Screening: Leveraging AI and adaptive optics to improve early detection of subclinical vascular/ neural changes, particularly in high-risk populations.
3. Mechanistic Insights: Elucidating the interplay between REC senescence, EndoMT and histone lactylation to identify druggable targets.
4. Therapeutic Innovation: Combining anti-inflammatory, anti-angiogenic, and metabolic modulators (e.g., H_2_S donors, heparanase inhibitors) with patient-tailored regimens. Clinical trials should prioritize interventions that address hyperglycemic memory and systemic comorbidities.
5. Patient-Centric Strategies: Enhancing adherence through tele-retina screening, lifestyle interventions, and educational programs to bridge gaps in care accessibility. By addressing these priorities, researchers and clinicians can transform DR management from reactive to proactive, preserving vision and improving quality of life for millions of diabetic patients.
